# The Role of S100A6 in Human Diseases: Molecular Mechanisms and Therapeutic Potential

**DOI:** 10.3390/biom13071139

**Published:** 2023-07-17

**Authors:** Fengguang Yang, Jinglin Ma, Daxue Zhu, Zhaoheng Wang, Yanhu Li, Xuegang He, Guangzhi Zhang, Xuewen Kang

**Affiliations:** 1 Department of Orthopedics, Lanzhou University Second Hospital, Lanzhou 730030, China; 120220901501@lzu.edu.cn (F.Y.); hexg18@lzu.edu.cn (X.H.); zhanggzh18@lzu.edu.cn (G.Z.); 2 The Second Clinical Medical College, Lanzhou University, Lanzhou 730030, China; 3 Orthopaedics Key Laboratory of Gansu Province, Lanzhou University Second Hospital, Lanzhou 730030, China; 4 School of Petrochemical Engineering, Lanzhou University of Technology, Lanzhou 730050, China

**Keywords:** S100A6, tumor, Alzheimer’s disease, leukemia, endometriosis, myocardial infarction

## Abstract

S100A6, also known as calcyclin, is a low-molecular-weight Ca^2+^-binding protein from the S100 family that contains two EF-hands. S100A6 is expressed in a variety of mammalian cells and tissues. It is also expressed in lung, colorectal, pancreatic, and liver cancers, as well as other cancers such as melanoma. S100A6 has many molecular functions related to cell proliferation, the cell cycle, cell differentiation, and the cytoskeleton. It is not only involved in tumor invasion, proliferation, and migration, but also the pathogenesis of other non-neoplastic diseases. In this review, we focus on the molecular mechanisms and potential therapeutic targets of S100A6 in tumors, nervous system diseases, leukemia, endometriosis, cardiovascular disease, osteoarthritis, and other related diseases.

## 1. Introduction

S100A6, also known as calcyclin, is a low-molecular-weight Ca^2+^-binding protein that contains two EF-hands belonging to the S100 protein family. The S100 protein family comprises more than 20 members, most of which encode genes, including S100A6, that are clustered on human chromosome 1q21 [1]. S100A6 was initially purified from Ehrlich ascites tumor cells [2]. S100A6 is highly conserved among species, containing 89 amino acid residues [3,4] in rats and mice and 90 amino acid residues [5,6] in humans and rabbits. S100A6 in chickens can be divided into two isomers, A and B, according to the presence of a lysine residue at the C-terminus [7]. S100A6 usually exists in the form of a homodimer, but heterodimers have also been reported [8,9].

**Figure 1:**
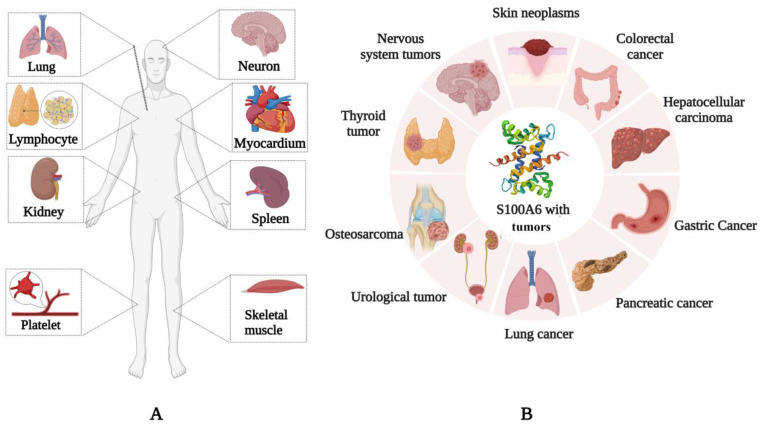
Status of S100A6 expression in mammalian normal tissues and human tumors. (**A**) S100A6 is ubiquitously expressed in various mammalian cells and tissues (such as skeletal muscle, myocardium, lung, kidney, spleen, neurons, platelets, and lymphocytes). (**B**) S100A6 has increased expression level in colorectal cancer, hepatocellular carcinoma, gastric cancer, cancer, pancreatic cancer, lung cancer, urinary tumor, osteosarcoma, and thyroid neoplasm. In other tumors, the expression level varies according to tumor type and stage.

S100A6 is expressed in various mammalian cells and tissues. S100A6 is expressed in the skeletal muscle, myocardium, lung, kidney, spleen [10], neurons [11], platelets [12], and lymphocytes (Figure 1A) [13], and it is upregulated in many tumors, such as lung [14], colorectal [15], and pancreatic cancers [16], as well as in melanoma [17] and hepatocellular carcinoma [18] (Figure 1B). It can regulate tumor invasion, proliferation, and migration and has the potential to become a therapeutic target. S100A6 can also be secreted by cells [19] and it is found in the extracellular matrix and some body fluids [20]. S100A6 is expressed in a variety of tissues and is closely related to its molecular function in specific sites or diseases.

In addition to being associated with tumors, S100A6 also plays an important role in many non-neoplastic diseases. S100A6 is present in various types of cells of the rat nervous system [21]. In Alzheimer’s disease (AD) patients and mouse models, S100A6 is concentrated in astrocytes surrounding the Aβ amyloid deposits of senile plaques [22]. S100A6 not only participates in the occurrence and development of Alzheimer’s disease but also plays different roles in neurological diseases such as amyotrophic lateral sclerosis [23] and epilepsy [24]. In the blood system, S100A6 is involved in the pathogenesis of MLL-AFF4-positive acute lymphoblastic leukemia (ALL) and the graft-versus-leukemia (GVL) effect after allogeneic hematopoietic stem cell transplantation [25,26,27]. Serum S100A6 protein is expected to be a diagnostic marker and therapeutic target for radiographic knee osteoarthritis (rKOA) in osteoarthritis [28,29]. In diseases of the cardiovascular system, S100A6 is involved in the structural repair and functional reconstruction of the heart after myocardial infarction as well as in the maintenance, repair, and reconstruction of the vascular intima [30,31,32,33]. In endometriosis, S100A6 is associated with the implantation and survival of ectopic endometrial stromal cells (euESCs) in the extrauterine environment [34]. In addition to these diseases, S100A6 has also been linked with cigarette-induced airway damage, liver fibrosis, and toxoplasma gondii infection [35,36,37]. Abnormal expression of S100A6 in many diseases is closely related to its pathogenesis and is a promising therapeutic target.

In this review, we mainly focus on the molecular mechanisms and potential therapeutic targets of S100A6 in tumors (colorectal, gastric, pancreatic, and lung cancers, hepatocellular carcinoma, urinary tumors, osteosarcoma, thyroid tumors, nervous system tumors, melanoma, etc.), nervous system diseases, leukemia, endometriosis, cardiovascular system diseases, osteoarthritis, and other related diseases.

## 2. S100A6 Association with Tumors

S100A6 has increased expression levels in colorectal cancer, hepatocellular carcinoma, gastric cancer, cancer, pancreatic cancer, lung cancer, urinary tumor, osteosarcoma, and thyroid neoplasm. In other tumors, the expression level varies according to tumor type and stage (such as nervous system tumors, skin tumors, etc.). S100A6 can be used as a biomarker for diagnosis and prognosis, and it also participates in the occurrence and development of a number of tumors through various intracellular mechanisms, affecting the progression and prognosis of tumors.

### 2.1. S100A6 in Colorectal Cancer

**Figure 2:**
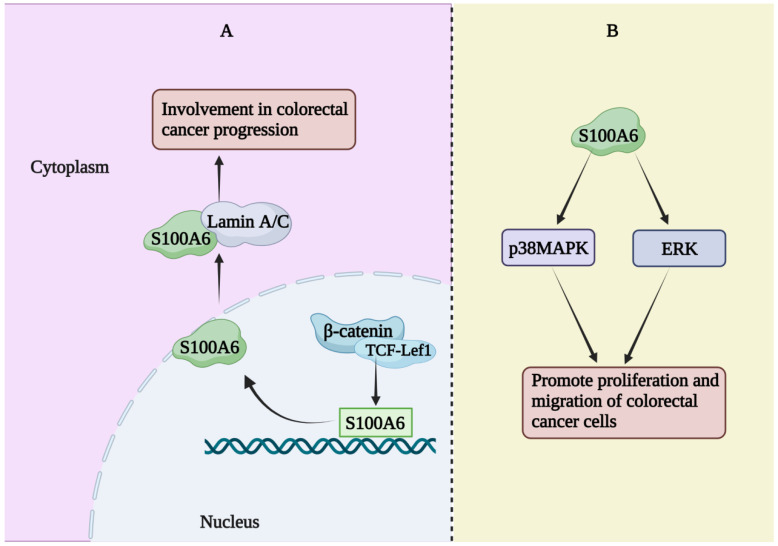
Function of S100A6 in colorectal cancer. (**A**) β-catenin and TCF-Lef1 co-transfection activated the promoter of S100A6, promoting the increase in S100A6 expression. S100A6 interacted with lamin A/C to promote the progression of colorectal cancer. (**B**) S100A6 can also promote the proliferation and migration of colorectal cancer cells by activating p38MAPK and ERK-signaling pathway.

The expression of S100A6 mRNA in advanced colorectal adenoma has been reported to be significantly higher than that in a healthy control group [38]. However, a single center prospective study showed a significant decrease in serum S100A6 and S100A11 levels, a significant increase in S100A8 levels, and no change in S100A9 levels in patients with colorectal cancer [39]. The expression of S100A6 has been reported to be increased in colorectal cancer tissues and to co-localize with β-catenin in colorectal cancer tissues as well as in metastatic SW620 and non-metastatic SW480 cells (human colorectal cancer cell lines) [40]. β-catenin and TCF-Lef1 were co-transfected to activate the S100A6 promoter, thereby increasing S100A6 expression. S100A6 can interact with lamin A/C (a protein known to be involved in colon cancer development) [40]. Therefore, interactions between these proteins may be involved in the progression of colorectal cancer. In addition, a study has demonstrated that S100A6 is necessary for Ca^2+^-dependent nuclear translocation of CacyBP/SIP in colon cancer [41]. Two members of the S100 family, S100A6 and S100A4, are believed to be involved in cancer invasion and metastasis. Studies have explored their role in human colorectal adenocarcinoma, indicating that S100A6 is involved in the process of human colorectal adenocarcinoma invasion. When cancer cells reformed glandular structure in the central site of metastatic nodules, S100A6 expression levels were reduced, while S100A4 expression did not appear to differ significantly between colorectal adenocarcinoma and control groups [42]. Similar results have been found in studies whereby S100A6, but not S100A4, was associated with tumorigenesis and human colorectal adenocarcinoma invasion/metastasis [43]. The average level of S100A6 in human colorectal adenocarcinoma was significantly higher than that in normal mucosa (approximately 2.4 times) and was significantly correlated with the Dukes tumor stage and lymphatic invasion, but not with other clinicopathological factors [44]. S100A6 was more abundant (based on staining) in the peripheral part of the adenocarcinoma than in the central part, while Ki-67 (a growth marker) was equally abundant in both parts. These results suggest that S100A6 is involved in the progression and invasion of human colorectal adenocarcinoma [44]. There was a statistically significant correlation between S100A6 expression and colon cancer progression [45]. The effects of S100A6 on the proliferation and migration of colorectal cancer cells are mediated through the ERK and p38MAPK pathways, and the regulation of these pathways may be used for the prevention and treatment of colorectal cancer [46]. S100A6 regulates the occurrence and progression of human colorectal cancer (Figure 2) and is a potential target for prevention and treatment.

### 2.2. S100A6 in Hepatocellular Carcinoma

A cDNA array with probes for 15,843 genes/clones detected differentially expressed genes in hepatocellular carcinoma (HCC) and non-cancerous liver tissues, and the expression of osteopontin and S100A6 in the two tissues differed by more than 10-fold, and they were only expressed in HCC [47]. Therefore, osteopontin and S100A6 are potential diagnostic markers and therapeutic targets for HCC [47]. The expression levels of S100A6 mRNA and protein in tissue samples detected by northern blotting and immunohistochemical staining, respectively, may also be a useful marker to distinguish cholangiocarcinoma (CC) from HCC [48]. The expression level of S100A6 in HCC tissues was higher than that in the adjacent non-tumor liver samples. S100A6 promotes the proliferation and migration of liver cancer cells by enhancing p53 ubiquitin-dependent proteasome degradation and ultimately regulates the expression of p21 [18]. In the human hepatoblastoma cell line HepG2, the transcription factor NF-κB helps activate S100A6 gene expression in response to TNF-α. TNF-α is a strong NF-κB activator required for hepatocyte proliferation during liver regeneration [49].

### 2.3. S100A6 in Gastric Cancer

The expression level of S100A6 in gastric cancer (GC) tissues and metastases is significantly higher than that in matched non-cancer tissues, and it correlates with the tumor size, depth of cell wall invasion, lymph node involvement, liver metastasis, vascular invasion, and tumor lymph node metastasis staging. S100A6 may hence be a promising diagnostic and prognostic marker and may be involved in the progression of gastric cancer [50,51,52]. Serum S100A6 levels in GC patients are also significantly higher than those in healthy controls, and they are closely related to lymph node metastasis, TNM stage, nerve invasion, and vascular invasion, in addition to being an independent predictor of overall survival [53]. The expression of S100A6 in gastric cancer is influenced by promoter methylation and histone H3 acetylation levels [52]. Studies have shown that increased S100A6 expression can promote the proliferation, invasion, and metastasis of gastric cancer cells by regulation of the expression levels of specific downstream regulatory factors (such as IL-8, CDK5, CDK4, MCM7, and Bcl2) [15,54]. The influence of S100A6 on the biological behavior of gastric cancer may also be related to the interaction between S100A6 and its ligand CacyBP/SIP [55], and further studies are needed to elucidate its mechanism of action.

### 2.4. S100A6 in Pancreatic Cancer

Current data indicate that S100A6 presents a hierarchical increase in the progression of pancreatic cancer, and it may hence be a biomarker for evaluating its malignant potential [56]. Measurement of S100A6 levels in pancreatic fluid may help detect early pancreatic cancer or identify individuals with high-risk lesions that may develop into pancreatic cancer [56]. EUS-FNA examination of S100A6 expression in pancreatic duct adenocarcinoma (PDA) shows high sensitivity and specificity for PDA diagnosis [16]. There are data showing that in pancreatic intraepithelial neoplasia (PanIN), as the PanIN grade increases, the percentage of S100A6-positive PanINs gradually increases, and the frequency and intensity of nuclear staining also increase [16]. Multi-factor analysis showed that nuclear S100A6 is an important independent indicator of survival, indicating that upregulation of S100A6 is an early event in the development of pancreatic cancer and that increased levels of nuclear S100A6 may affect clinical outcomes [16]. There was a strong correlation between high levels of S100A6 in the cytoplasm of pancreatic cancer cells and the presence of annexin 2 on the plasma membrane. Loss of S100A6 is accompanied by decreased levels of annexin 2, which leads to a significant decrease in pancreatic cancer cell viability. This may help explain the mechanism of S100A6 expression in pancreatic cancer invasion and metastasis [57]. Although S100A6 has been shown to be associated with the diagnosis and prognosis of pancreatic cancer, its potential mechanism in the occurrence and development of pancreatic cancer remains to be elucidated.

### 2.5. S100A6 in Lung Cancer

S100A6 and S100A2 levels are significantly elevated in the serum of patients with early-stage non-small cell lung cancer (NSCLC). Receiver operating characteristic (ROC) analysis showed that the S100A2 level could distinguish patients with non-small cell lung cancer from healthy controls (AUC (area under the ROC curve) = 0.646). S100A6 was also associated with NSCLC (AUC = 0.668). In addition, the levels of both proteins could be used to distinguish stage I/II NSCLC patients from healthy controls (AUC = 0.708 for S100A2 and 0.702 for S100A6). S100A6 and S100A2 may hence be potential biomarkers for NSCLC [58]. Multivariate analysis using a Cox regression model showed that a high expression of S100A6 is an independent adverse prognostic factor in lung squamous cell carcinoma (SCC), suggesting that S100A6 may be a drug target for SCC [59].

S100A6 is also associated with progression and/or invasion of lung adenocarcinoma, particularly bronchioloalveolar carcinoma (BAC). Immunohistochemistry can be used to evaluate the malignancy of BAC component adenocarcinoma [60]. In Calu-6 lung cancer cells, overexpression of S100A6 reduces cell proliferation, migration, and invasion, while enhancing cell apoptosis [14]. Therefore, at the cellular level, S100A6 appears to be a tumor suppressor gene in lung cancer [14]. However, almost the opposite results have been obtained. In vivo and in vitro studies have shown that S100A6 overexpression in NSCLC can promote the proliferation, invasion, migration, and angiogenesis of lung cancer cells by inhibiting p53 acetylation. Overexpression of miR-193a can reverse these effects by decreasing the expression of S100A6 (the target of miR-193a is S100A6) [61]. Similarly, in NSCLC, immunohistochemistry has revealed that the expression of S100A6 is negatively correlated with the expression of p53. Compared with S100A6-negative patients, S100A6-positive patients tended to have longer survival (*p* = 0.07), and S100A6 has been proposed as an independent prognostic factor for increased survival [62]. Why is lung cancer diagnostics so difficult to accomplish and why are there so many inconsistencies? Can it be associated with various post-translational modifications of S100A6 in lung cancer, which makes the role of S100A6 so putative and enigmatic? Are there potentially various PTMs of S100A6 instead of ‘messed’ expression patterns amongst NSCLC, SCC, lung adenocarcinoma, etc.? Ultimately, additional experiments will be required to further explore and verify its role.

It is gratifying to note that a study using tissue microarray technology screened hundreds of tumor specimens from eight different tumor types in patients, and it was found that RAGE (advanced glycation end-product receptor) is rich in expression in breast and lung tumor tissues. At the same time, it was also found that S100A4 and S100A6 have a certain correlation with the expression of RAGE. This suggests that RAGE-mediated signal transduction may play a role in the development of these specific cancers [63]. Due to the progress made in tumor treatment with anti-RAGE, the role of RAGE/S100 family proteins in the occurrence and development of lung cancer deserves attention and may become a potential therapeutic target.

### 2.6. S100A6 in Urological Tumors

Protein and mRNA levels of S100A6 in pathological tissues of renal clear cell carcinoma (ccRCC) and six renal cell lines (Kaki-2, 769-P, 786-O, ACHN, Kaki-1, and SN12-PM6) have been reported to be elevated [64]. The high expression of S100A6 may promote the metastasis of ccRCC by enhancing the migration and invasion ability of tumor cells. In addition, quantitative expression of S100A6 mRNA in tumor tissues is an independent risk factor and can be used as a biomarker for the risk of ccRCC metastasis at stages T1–T2 [64]. In addition, studies have found that the level of S100A6 in ccRCC tissues is related to the pathological characteristic grade and clinical stage of ccRCC patients. Moreover, inhibiting the expression of S100A6 can inhibit cell proliferation in vitro and tumor growth in vivo [65]. Gene expression profile analysis has shown that S100A6 has a new function of inhibiting apoptosis, and a relationship between S100A6 and the pro-inflammatory chemokine CXCL14 was found. Therefore, the S100A6/CXCL14-signaling pathway is considered a potential therapeutic target for ccRCC [65].

A major challenge in muscular invasive urothelial carcinoma (UC) is the identification of biomarkers that can predict disease prognosis and treatment response after a cystectomy. Although there are data showing that VEGFR2 expression is significantly associated with disease recurrence risk and overall survival in patients with urothelial carcinoma treated with cystectomy, there is a significant difference in survival of over two years in patients with low S100A6 expression compared to those with high S100A6 expression [66]. S100A6 may be related to the survival and prognosis of these patients. Unlike ccRCC, S100A6 expression is often absent in prostate cancer, and the possible mechanism is hypermethylation of the promoter region [67]. There is data indicating that loss of S100A6 protein expression is common in the progression of prostate cancer and may occur in the early stages. The mechanism of its lack of expression may be related to hypermethylation at the CpG site. In the basal cells of benign glands, the expression of S100A6 is significantly increased. This may become a useful diagnostic marker [68]. The expression of S100A6 in urinary tumors varies from high to low, and its mechanism of action may be different. Further mechanistic studies are expected to reveal the role of S100A6 in urinary tumors.

### 2.7. S100A6 in Osteosarcoma

Although osteosarcoma (OS) is the most common primary malignant tumor in bone, its molecular mechanism is not completely clear. S100A6 is expressed at significant levels in human OS cell lines and may promote the proliferation of osteosarcoma cells, at least partly by promoting cell cycle procession, preventing cell apoptosis, and inhibiting osteoblastic differentiation [69]. Although most osteosarcoma patients have metastatic or micrometastatic lesions, less than 15% have clinically detectable metastatic disease at the time of presentation [70]. Therefore, there is an urgent need for markers that can accurately identify existing metastatic disease or predict future metastatic disease in patients with osteosarcoma. Studies have shown that clinical evidence of metastasis tends to decrease with increasing S100A6 staining. S100A6 overexpression in osteosarcoma reduces cell motility and unanchored growth on collagen gels. This suggests that loss of S100A6 expression is related to the metastatic phenotype [70]. The same research team explored this using siRNA knockdown of S100A6 in four commonly used human osteosarcoma cell lines, followed by measurement of cell adhesion, migration, and invasive properties [71]. It was found that knockdown of S100A6 expression inhibited cell adhesion and promoted cell migration and invasion. In contrast, S100A6 overexpression enhanced cell adhesion and inhibited cell invasion. These results suggested that S100A6 may inhibit osteosarcoma metastasis by promoting cell adhesion and inhibiting cell motility and invasion. Therefore, S100A6 can be considered to be a potential marker of human osteosarcoma with prognostic value for identifying patients without metastasis [71].

### 2.8. S100A6 in Thyroid Tumors

Accurate diagnosis of thyroid tumors is challenging. The search for new molecular diagnostic markers through various technical methods is a promising approach that can be used as a potential supplement to conventional diagnosis. A proteomic analysis has shown that the expression of S100A6 in papillary thyroid carcinoma (PTC) was significantly higher than that in other tumor groups and normal tissues. Immunohistochemical analysis showed that the cytoplasmic staining of PTC was significantly stronger than that of follicular tumors, and the proportion of nuclear staining was significantly higher in PTC than in follicular tumors. This supports the notion that S100A6 is involved in the occurrence and development of thyroid tumors and that it can contribute to the differential diagnosis of follicular thyroid tumors and PTC [72]. Another study used matrix-assisted laser desorption/ionization (MALDI) imaging mass spectrometry (IMS) to compare the proteomic characteristics of metastatic and non-metastatic papillary thyroid cancers. It was found that the overexpression of thioredoxin and S100A10 and S100A6 proteins were highly correlated with lymph node metastasis of PTC and that this can be used for risk stratification of PTC metastatic potential [73]. In addition, some studies have found that S100A2 and S100A6 are involved in some events of PTC progression [74].

### 2.9. S100A6 in Nervous System Tumors

Neurothekeoma is a superficial tumor derived from the nerve sheath and has been described as a cell and mucus type. In recent years, it has become increasingly apparent that there is no differentiation of the nerve sheath into cell types. This subtype is easily confused with melanoma, leading to misdiagnoses. It is important to note that studies have found that the combination of S100A6’s strong immunoreactivity in the proliferation of spindle cells of nesting dermis and the lack of S100 protein or keratin support the diagnosis of cellular neurilemmoma [75]. The combination of the S100 protein family (including S100A6), galectin-3, and its ligand profile (through a decision tree) is helpful for differential diagnosis between benign and atypical meningiomas. Moreover, the galectin-3 binding site and S100B may play a role in the invasion of atypical meningiomas [76]. S100-positive supplementary cells are also an important feature of the olfactory neurolastoma [77]. It has also been shown that S100A6 and Ca^2+^ are important regulators of CacyBP/SIP phosphatase activity and ERK1/2-Elk-1-signaling pathway by inhibiting the phosphorylation of threonine 184 on CacyBP/SIP in NB2a NB cells [78].

In addition to the above, the study also found that four members of the S100 family (S100A2, S100A4, S100A6, and S100A10) were upregulated in multiple medulloblastoma cell lines after treatment with DNA methyltransferase inhibitors (50-aza-20-deoxycytidine) [79]. In addition, S100A6 hypermethylation is significantly associated with aggressive large cell/anaplastic morphophenotype. In contrast, in a considerable proportion of primary tumors and cell lines, S100A4 showed evidence of hypomethylation compared to normal cerebellum before metastasis, which may be the reason for its increased expression [79]. The above data indicate that the epigenetic imbalance of multiple S100 family members in the progression of medulloblastoma is closely related to the complex pattern of cerebellar S100 gene somatic cell methylation, which may also be the cause of medulloblastoma, with potential clinical value.

### 2.10. S100A6 in Melanoma

S100A2, S100A4, and S100A6, which are three members of the S100 gene family, are believed to be involved in the development and metastasis of cancer. The mRNA expression levels of these three genes were measured in 45 metastatic melanoma tumors and 20 benign nevi. Although S100A2 mRNA was not expressed in all metastatic tumors and the expression level was low in the six cell lines established by primary melanoma, the expression level in all nevi was medium to high. This suggests that loss of S100A2 gene expression may be an early event in the development of melanoma. The expression of S100A6 in melanoma metastasis was significantly correlated with the survival time of patients and the corresponding primary tumor thickness. There was no relationship between S100A4 gene expression and the clinical parameters of malignant melanoma. This result suggests that evaluation of the potential value of using S100A2 and S100A6 expression levels as clinical management markers for melanoma has ample merit [80]. Unlike the typical Spitz nevus with strong and diffuse S100A6 staining, the small spindle cells of pigmented spindle cell nevus usually exhibit patchy or incomplete staining. However, in melanocytic tumors composed of small spindle cells, patchy S100A6 staining should not be interpreted as evidence supporting the diagnosis of melanoma [81]. Spindle cell melanoma is a rare melanoma that is difficult to diagnose clinically and histopathologically from various non-melanocytic spindle cell tumors. Japanese researchers have reported a case of amelanotic melanoma that contained spindle cells positive for c-Kit and S100A6 staining. Therefore, the use of c-Kit and S100A6 may help improve the diagnosis [17].

Recent studies have shown that RAGE is a multi-ligand receptor, which participates in the progress of melanoma by promoting tumor growth [82,83]. However, the mechanism of RAGE activation in melanoma is still unclear. Some studies found that overexpression of RAGE in melanoma cell lines not only led to a significant increase in migration rate, but also led to a decrease in proliferation rate. In vivo tumor formation experiments in mice showed that RAGE-overexpressing cells produced tumors faster than the control group, and tumor protein extract analysis showed that the levels of RAGE ligands S100B, S100A2, S100A4, S100A6, and S100A10 were increased in RAGE-overexpressing tumors [84]. Animal studies on anti-RAGE antibodies have shown that RAGE blocking leads to reduced tumor growth and metastasis of melanoma [85]. This indicates that the RAGE/S100 family proteins are closely related to the progress of melanoma.

### 2.11. S100A6 in Skin Tumors

The skin is the largest organ in the human body. It covers the entire body surface and can be divided into two layers: the epidermis and dermis. Generally, the skin is directly in contact with the external environment. The epidermis is located on the surface of the skin and belongs to the stratified squamous epithelium, which can be divided into cuticle and germinal layers. The dermis is located underneath the epidermis and contains dense connective tissue. There are many skin tumor diseases such as syringoma, lipoma, leiomyoma, melanocytic nevus, basal cell carcinoma, squamous cell carcinoma, and malignant melanoma. Malignant melanoma mostly occurs in the skin but can also occur in the mucosa and viscera. We introduced this separately in the previous section. In this section, we focus on other skin tumor diseases and S100A6 expression.

Studies have shown that S100A6 is expressed in squamous cell carcinoma (SCC) but not in basal cell carcinoma (BCC) and partially in microcystic adnexal carcinoma (MAC). Tumors with ductal differentiation expressed S100A6 differently, and the two eccrine adenomas showed the strongest staining. In addition to malignant peripheral nerve sheath tumors, S100A6 has a high expression level in malignant spindle cell tumors [86]. This also shows that S100A6 expression can distinguish SCC from BCC, MAC from BCC, and eccrine adenoma from other adnexal tumors [86]. As previously mentioned, the expression of S100A2, S100A3, and S100A6 in pilomatrixoma is conducive to determining the source of cells in abnormally differentiated tissues [87]. According to the unique immunohistochemical staining mode of S100A6 antibody in columnar leiomyoma (LM), angioleiomyoma (ALM), and skin leiomyoma (LMS), it is helpful for the differential diagnosis between them: weak or no S100A6 staining supports the diagnosis of LM, whereas strong positive staining supports the diagnosis of LMS [88]. In addition, some studies have shown that the expression of S100A6 is significantly related to the malignant transformation of epidermal tumors, and the joint expression of S100A6 and MMP9 is related to the development of SCC [89].

### 2.12. S100A6 in Other Tumors

S100A6 is not only widely involved in the occurrence and development of the above tumors but is also related to several other tumor diseases. Since there have only been a handful of relevant studies to date, we classify them here. Western blot analysis and real-time PCR results have shown that S100A6 protein and mRNA levels are reduced in vitro and in vivo upon carcinogenesis of oral squamous cell carcinoma (OSCC) [90]. Doxorubicin has been shown to induce the expression of miR-21-5p in mesenchymal stem cells and their derived exosomes (MSC-Exo) and then regulates S100A6 to induce breast cancer cell resistance to chemotherapy [91]. Proteomic analysis of tumor interstitial fluid and in vitro and in vivo experiments have shown that S100A6 is highly expressed in cholangiocarcinoma tissues and promotes the proliferation of cholangiocarcinoma cells by activating the p38/MAPK pathway. Therefore, S100A6 can be used as a marker for screening and prognostic analysis as well as a potential target for the treatment of cholangiocarcinoma [92,93]. However, no abnormal S100A6 expression was detected in the serum of patients with cholangiocarcinoma [94]. Loss of S100A6 protein expression has been shown to be common in prostate cancer progression, which may occur in the early stages. The mechanism of its loss of expression may be related to the hypermethylation of CpG sites. The expression of S100A6 was significantly increased in the basal cells of benign glands. This may serve as a useful diagnostic marker [68]. S100A6 expression in human nasopharyngeal carcinoma tissues and cell lines was significantly higher than that in matched normal peritumoral tissues and normal nasopharyngeal epithelial cell lines, and it was associated with advanced N stage, local failure, and disease progression, in addition to being an independent prognostic factor of local recurrence-free survival and progression-free survival [95]. It can also promote the progression of nasopharyngeal carcinoma by activating the p38MAPK signaling pathway [95]. In cervical cancer, S100A6 promotes and mediates the malignant potential of cancer cells by activating the PI3K/Akt-signaling pathway, thereby selectively activating the ability to metastasize and epithelial–mesenchymal transition [96]. Compared with patients with early ovarian cancer, S100A6 in the serum of patients with advanced ovarian cancer was significantly increased and was related to the experimental tumor burden and the clinical disease stage. These data suggest that S100A6 in combination with other biomarkers may be useful for detecting and/or monitoring ovarian cancer [97]. Granulosa cell tumors (GCT) are rare mesenchymal soft-tissue tumors that can occur throughout the body. Most are benign, but approximately 2% are malignant. Their differential diagnosis is difficult [98]. A case report found that S100A6 was expressed in Schwann cells of both primary and metastatic tumors. S100A6 may be a marker for the differential diagnosis between benign and malignant GCT, but a larger cohort study is needed to verify this possibility [99].

S100A6 is differentially expressed in many tumors, and it participates in tumor progression through the corresponding mechanism. It has value as a diagnostic, for differential diagnosis and as a prognostic marker, and it may also become a potential therapeutic target. It is worth noting that S100A6 can be valuable as an auxiliary marker in combination with other specific proteins, including other members of the S100A family. It is unknown whether S100A6 has a common mechanism of action in different tumors. However, it has been found that chromosome rearrangement often occurs at 1q21 during tumor transformation, S100 gene expression is out of balance, and a modification mode is formed in tumor tissue [100]. Inhibition of S100A6 is considered to be a mechanism by which p53 inhibits tumor cell proliferation, and the insufficient inhibition of the p53 mutant on S100A6 may be the reason for its overexpression and cell cycle imbalance in cancer tissue [101]. RAGE is a member of the immunoglobulin family. Its interaction with ligands can trigger downstream signal transduction and induce inflammatory reactions related to diabetes, inflammation, cancer, cardiovascular disease, and various other human diseases [102]. A study has explored effective targets for human malignant tumors related to the RAGE-S100A6 complex and conducted structure-based virtual screening using the ZINC15 database, with the potential to develop new drugs for the treatment of related tumors [102]. S100A6 is widely involved in the progression of tumor lesions, playing a variety of roles in this process. Further mechanistic research may help reveal its potential molecular mechanism.

## 3. S100A6 in Nervous System Diseases

An immunohistochemical method was used to detect the distribution of S100A6 in the nervous system of rats. It was found that S100A6 was mainly distributed in (1) specific areas of the limbic system (such as the basolateral amygdaloid nucleus, the ventral apex of the CA1—subglobular marginal area, the entorhinal cortex, and the paracortex), and most of them were identified as subpopulations of pyramidal cells; (2) olfactory receptor cells, olfactory nerve fibers, and terminals in the olfactory bulb; (3) some tracts of the hindbrain and spinal cord (such as the trigeminal spinal tract, single bundle, dorsal root fiber, and the tract of Lissauer and its terminals (such as the trigeminal main sensory nucleus, trigeminal spinal nucleus, single bundle nucleus, marginal area, substantia gelatinosa, and dorsal horn proper sensory nucleus)), and some sensory neurons originating from the dorsal root and trigeminal ganglion; (4) the white matter of the brain (such as the corpus callosum, cingulate, outer membrane, inner capsule, and chorionic membrane of the hippocampus) and the astrocyte subsets around the ventricle; (5) ependymal cells, especially around the central canal; and (6) Schwann cells [21]. In some studies, immunocytochemistry was used to detect the temporal and spatial expression patterns of S100B and S100A6 in the hippocampus, entorhinal cortex, and occipital cortex of normal human fetuses. The number of S100A6-positive cells in the detected brain region was less than the number of S100B-positive cells. The immunoreactivity of S100A6 in some pyramidal neuron-like cells and some glial-like cells in the pyramidal and molecular layers of the hippocampus increased in the second trimester of pregnancy and decreased in late fetal and elderly adult samples. Weak staining of S100A6-positive cells was also observed in the olfactory cortex and the cortex of elderly adults throughout the pregnancy. The S100A6 immune response in the fetal occipital cortex is weak. The difference in temporal and spatial expression between the two may indicate that they play different roles at different stages of development [103].

In adult rodents, proliferating cells in the subventricular area migrate tangentially to the olfactory bulb and become neurons in the olfactory bulb. Immunocytochemical staining has revealed that astrocytes containing S100A6 are localized in the tangential migration pathway, which may be related to this process [104]. S100A6 is also highly expressed in neural stem cells and astrocyte precursors in the subgranular zone of the adult mouse hippocampus, which may contribute to the generation of astrocytes in the hippocampus [105]. Under conditions of stress, the expression of S100A6 in the nervous system of mice changed, and the expression in most structures (such as the olfactory bulb, hippocampus, hypothalamus, etc.) decreased. Although the mechanism remains unclear, it nonetheless shows that S100A6 is involved in the stress response [106]. In a rat epilepsy model induced by amygdala stimulation, S100A6 was widely and persistently upregulated in the cortex, hippocampus cortex, CA1 area, and GFAP-positive astrocytes (located in the hippocampus and cortex), which may be related to the proliferation of astrocytes [24]. However, after tetanizing or low-frequency stimulation, the mRNA level of S100A6 in the hippocampus of rats was low and exhibited little change [107,108]. However, in the rat traumatic brain injury (TBI) model, S100A6 protein and mRNA levels in the hippocampus decreased 1 h after brain injury and returned to the original level on the 14th day. This decrease may be one of the early events that cause secondary cognitive decline after TBI. The subsequent recovery of the expression level may be closely related to cognitive improvement. This indicates that S100A6 may participate in the degeneration and regeneration of neurons following brain injury [109]. Some studies have reported expression of Ascl1, Casp3, and S100A6 in the hippocampus, prefrontal cortex, and cerebellum of rats during water maze spatial memory performance. Compared with the control group, the expression of three genes in the active learning group increased in the prefrontal cortex, Casp3 and Ascl1 increased in the hippocampus, while only S100A6 increased in the cerebellum [110]. These findings suggest that S100A6 may be involved in memory acquisition in the hippocampus and its integration into the prefrontal cortex and cerebellum.

Amyotrophic lateral sclerosis (ALS) is a neurodegenerative disease characterized by selective degeneration of motor neurons. Astrocyte proliferation is one of its early pathological changes, including sporadic amyotrophic lateral sclerosis (SALS), accounting for 90–95% of cases, and familial amyotrophic lateral sclerosis (FALS), accounting for 5–10% of cases [111]. FALS is an autosomal phenotypic disease, its onset is age-dependent, and it is closely related to mutation of the homodimeric enzyme Cu/Zn superoxide dismutase 1 (SOD1). Transgenic mice expressing the human SOD1^G93A^ mutant gene are commonly used as models of the disease. In a transgenic mouse model, it was found that S100A6 expression in reactive astrocytes in several regions (including 12 pairs of nerve roots) of the brain stem increased [23] and that these astrocytes may be located near denatured motor neurons [111]. Although electrophysiological data suggest that selective lesions of motor neurons may be related to their calcium buffering capacity [23], there is still a lack of data in regard to whether this is related to S100A6 overexpression in ALS. In addition, some studies have shown that S100A6 in astrocytes may participate in neuronal damage in response to extracellular glutamate under pathophysiological conditions [112].

Alzheimer’s disease (AD) is a progressive neurodegenerative disease [113,114]. Excessive accumulation of extracellular amyloid-β (Aβ) peptides in the brain is an important feature of AD [115,116]. S100A6 protein has been found to be uniformly upregulated in the white matter of AD patients and mouse models. However, the S100A6 immune response in gray matter is almost exclusively concentrated in astrocytes surrounding the Aβ amyloid deposits of senile plaques [22]. Chronic zinc exposure leads to Aβ deposition and increased S100A6 expression in mouse models, which can be reversed by zinc chelation [117]. The possible mechanism is that Zn^2+^ is highly concentrated in the Aβ amyloid deposits of AD and S100A6 has a high affinity for Zn^2+^. S100A6 competitively combines with Zn^2+^ to make Aβ amyloid deposit depolymerization [22]. In brain sections with AD-like lesions, increased S100A6 levels promote Aβ depolymerization and decrease the zinc content in Aβ plaques. Therefore, zinc chelation may be a potential treatment strategy for AD [117].

**Figure 3:**
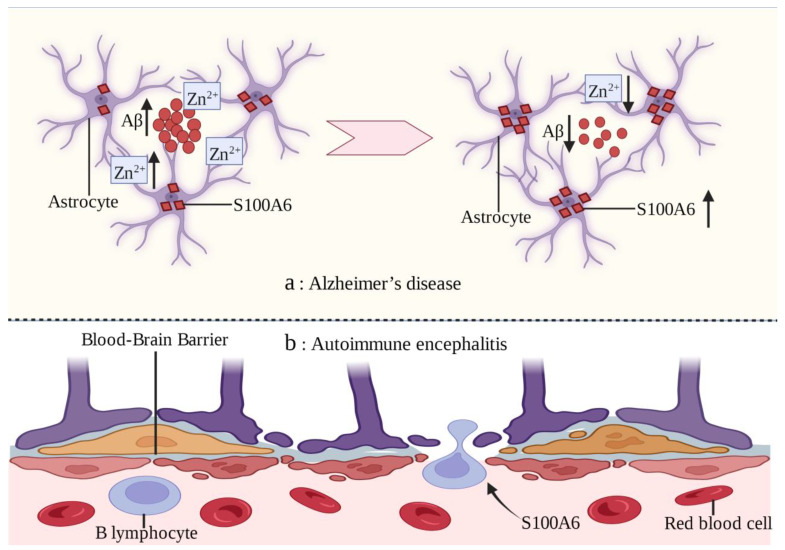
Mechanism of action of S100A6 in Alzheimer’s disease and autoimmune encephalitis. (**a**) In patients with Alzheimer’s disease, S100A6 is concentrated in the gray matter in astrocytes surrounding the Aβ amyloid deposits of senile plaques, and chronic zinc exposure leads to Aβ deposits and increased S100A6 expression. However, the increase in S100A6 level can promote the depolymerization of Aβ and reduce the zinc content of Aβ plaques. (**b**) S100A6 can promote B lymphocytes to penetrate the blood–brain barrier, which is related to the pathogenesis of autoimmune encephalitis.

Autoimmune encephalitis (AE) is a serious nervous system disease in which the brain becomes the target of immune disorders [118]. Infiltration of autoantibodies and B lymphocytes through the blood–brain barrier (BBB) layer is an important feature of AE [119]. The in vitro leukocyte transendothelial migration model confirmed that S100A6 can promote B lymphocytes to penetrate the BBB, which is similar to the in vivo mechanism [120] (Figure 3). Further exploration of the underlying mechanism of this phenomenon will help improve the understanding of AE and identify new therapeutic targets.

S100A6 is widely expressed in the nervous system and plays a variety of roles in the occurrence and development of various nervous system diseases. With further study of S100A6 in related diseases, it is expected to become a therapeutic target for some diseases. S100A6 and its ligand play a variety of roles in nervous system diseases [121]. Interaction with ligands may also be an important mechanism by which S100A6 plays a role in nervous system diseases.

## 4. S100A6 in Leukemia

**Figure 4:**
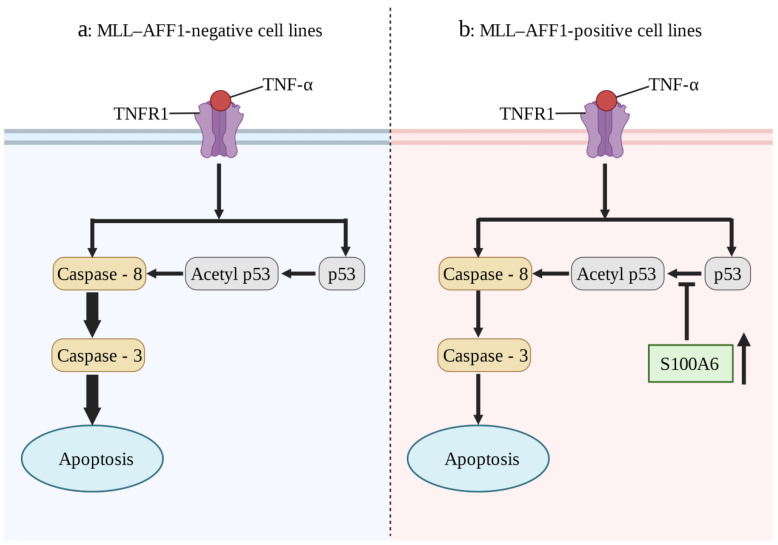
The expression of S100A6 affects the effect of TNF-α on ML-Aff1-positive ALL cell lines. (**a**) The effect of TNF-α on MLL- AFF1-negative cell lines. TNF-α may promote apoptosis of leukemia cells through caspase-8/caspase-3 pathway or p53/caspase-8/Caspase-3 pathway. (**b**) Mechanism of anti-TNF-α effect in MLL- AFF1-positive cell lines. MLL-AFF1-positive ALL cell lines seem to resist the TNF-α/p53/caspase 8/caspase3 pathway by up-regulating S100A6 expression. (Adapted from reference [28]).

S100A6, as a key regulator of hematopoietic stem cells [122], plays an important role in the pathogenesis of a number of blood system diseases, especially in MLL-AF4-positive leukemia. In 32Dc (a murine interleukin-3-dependent cell line), overexpression of MLL-AF4 or mutant Flt3-TKD (Fms-like tyrosine kinase 3 (Flt3) gene mutation in the tyrosine kinase domain (TKD)) alone failed to result in an IL-3-dependent proliferation capacity. However, when Flt3-TKD and MLL-AF4 were expressed together, an IL-3-independent proliferation capacity was observed [25]. This suggests that the synergistic enhancement of S100A6 expression by MLL-AF4 and Flt3-TKD plays a key role in MLL-AF4 leukemia. The prognosis of allogeneic hematopoietic stem cell transplantation for mixed lineage leukemia (MLL)-AFF1 (MLL-AF4)-positive acute lymphoblastic leukemia (ALL) is generally poor, which may be due to the resistance to the graft-versus-leukemia (GVL) effect [26]. Tumor necrosis factor-α (TNF-α) plays an important role in GVL. Overexpression of S100A6 in MLL-AFF1-positive ALL cell lines inhibited the p53 caspase 8/caspase 3 pathway and attenuated TNF-α-mediated apoptosis (Figure 4) [27]. Inhibition of S100A6 expression in MLL-AFF1-positive ALL mouse models can significantly prolong survival [123]. Therefore, inhibition of S100A6 expression in combination with allogeneic hematopoietic stem cell transplantation is a promising target for the treatment of MLL-AFF1-positive ALL. At present, drug studies are seeking to elucidate this mechanism. For example, in KMT2A/AFF1-positive transgenic mice, the antiallergic drug amlexanox can inhibit the expression of S100A6 in the presence of TNF-α and can also enhance the tumor immunity of model mice, reduce the incidence rate, and extend the survival period [124]. Although it has not yet entered clinical transformation, it is an approach that has ample therapeutic potential.

## 5. S100A6 in Cardiovascular Diseases

An abundance of published data have shown that S100A6 plays an important role in cardiovascular diseases. It has been reported that after treatment with several nutrients (including platelet-derived growth factor, the α1-adrenergic agonist phenylephrine, and angiotensin II), S100A6 expression increases around the infarcted area of rat hearts and in cultured neonatal rat cardiomyocytes, and transfection of S100A6 into neonatal rat cardiomyocytes can inhibit the promoter activity of several genes [125]. This suggests that S100A6 may be involved in the regulation of cardiac differentiation. In the serum of patients with acute coronary syndrome, the expression of S100B, S100A6, S100P, and their receptors, sRAGE, increased. The expression of the first three entities is also closely related to the size of the myocardial infarction [126]. The S100 family of proteins, especially S100A6, may hence play a key role in heart development and heart disease by binding to their ligands.

Epidemiological data show that compared with women, men have a higher prevalence of cardiovascular diseases and mortality, and cardiac complications occur earlier and progress faster [127,128]. In males, with an increase in age, myocardial cells are gradually lost, and the remaining myocardial cells become hypertrophic. However, the total number of cardiac myocytes in women remains relatively stable throughout their lives [129]. Studies on animal models of cardiomyopathy have shown that activation of the CB1 receptor (cannabinoid receptor) is related to myocardial cell injury, increased collagen deposition, and excessive growth of myocardial cells, whereas the CB2 receptor (cannabinoid receptor) is related to heart protection, anti-fibrosis, and anti-myocardial hypertrophy [130,131,132,133,134,135,136,137]. Through transmembrane channels (L-type Ca^2+^ channels, T-type Ca^2+^ channels, and Na^+^/Ca^2+^ exchangers) and calcium oscillations, cannabinoids regulate the calcium current between the sarcoplasmic reticulum and cytoplasm, thereby regulating the calcium content in myocardial cells and affecting the contraction and relaxation of the myocardium [138,139]. The regulation of calcium homeostasis in the myocardium is inseparable from the regulation of calcium-binding proteins, especially the S100 family proteins [140]. Increasing evidence indicates that S100A6 plays an important role in heart system diseases [30,31,125,126,141]. Some studies have conducted immunostaining and comparative analysis of cannabinoid receptors (CB1 and CB2), S100A6, and CacyBP/SIP in the myocardium of elderly men and women and found that the expression and distribution of cannabinoid receptors (CB1 and CB2), S100A6, and CacyBP/SIP genes in the human heart are sex- and age-dependent [142]. Similar studies have compared the distribution of cannabinoid receptors (CB1 and CB2), Apelin, and S100A6 proteins in the hearts of healthy women of various ages. There were significant differences in the immunoreactivity and distribution of CB1, CB2, Apelin, and S100A6 in the two age groups above and below 50 years of age, which indicates that they may play different roles in the heart at different ages [143].

In S100A6-overexpressing cardiomyocyte-specific transgenic mice (S100A6-MHC-tTA), after permanent coronary ligation, hypertrophy of cardiomyocytes was reduced and less interstitial fibrosis and cardiomyocyte apoptosis were observed, which helped preserve the remaining cardiac function. S100A6 may be a potential therapeutic target for early ventricular remodeling after myocardial infarction [30]. In addition, S100A6 is helpful in improving ischemia-reperfusion injury and reducing mortality. S100A6 overexpression can reduce myocardial cell apoptosis, myocardial hypertrophy, and infarct area as well as improve left ventricular systolic function after ischemia/reperfusion [31]. This further supports the notion that S100A6 is a therapeutic target for myocardial infarction. TNF-α can induce cardiomyocyte apoptosis, whereas S100A6 can prevent TNF-α from inducing cardiomyocyte apoptosis by interfering with p53 phosphorylation [144]. Moreover, S100A6 may also play a role in myocardial protection in patients with hypertension [141].

S100A6 expression was also observed in vascular endothelial cells, and it was present in the cytoplasm and the nucleus. Loss of S100A6 expression reduces the expression of cyclin-dependent kinase 1, p-cyclin-dependent kinase 1, cyclin A1, and cyclin B genes, affects cell cycle progression, increases G2/M phase cell cycle arrest, and leads to endothelial cell senescence [32]. S100A6 has also been shown to be an inhibitor of STAT1 (signal transducers and activators of transfer 1), which plays an important role in the process of re-endothelialization of damaged blood vessels [33]. In general, S100A6 participates in the occurrence and development of cardiovascular system diseases, plays an important role in cardiac structural repair and functional reconstruction after myocardial infarction, and regulates the endothelial cell cycle, thus playing a key role in maintaining the integrity of the vascular intima in health and disease, as well as in repair and reconstruction.

## 6. S100A6 in Other Diseases

In addition to participating in the above-mentioned diseases, S100A6 is also related to the pathophysiological processes of several other diseases. S100A6 is closely related to the occurrence and development of endometriosis. Endometriosis is a common gynecological disease characterized by estrogen dependence. The presence and growth of endometrial tissue outside the uterine cavity are important features. Although it is a benign disease, it is characterized by ectopic invasion and other malignant tumors. Approximately 10% to 15% of women of reproductive age suffer from the disease, which often leads to lower abdominal pain and infertility [145] and thus has a serious impact on quality of life [146]. It has been found that the transcription levels of CD63, S100A6, and *GNB2L1* in the tissues of ectopic endometrium are higher than those in normal endometrium (regardless of endometriosis), independent of the menstrual cycle stage. These three genes are thought to be involved in cell proliferation, apoptosis, and angiogenesis, which interfere with the homeostasis of cells in the ectopic endometrium, thus contributing to their implantation and survival in the extrauterine environment [34].

**Figure 5:**
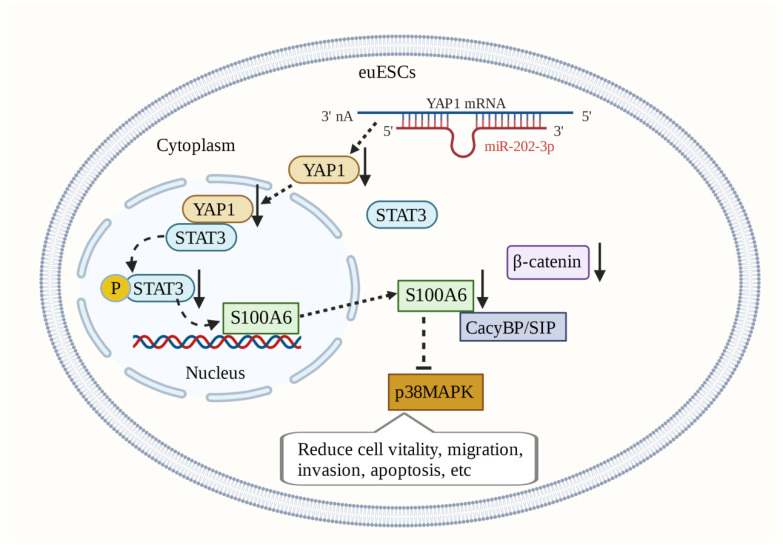
In ectopic endometrial stromal cells (euESCs), miR-202-3p targets YAP1 to reduce STAT3 mediated S100A6, thus preventing the progress of Endometriosis. S100A6 expression and β-catenin are synergistic. S100A6 can also interact with CacyBP/SIP to regulate the activity of the p38MAPK pathway and affect the activity, apoptosis, invasion, and migration of euESCs.

Silencing S100A6 expression in ectopic endometrial stromal cells (euESCs) reduces p38MAPK activity in euESCs and inhibits cell viability, migration, and invasion. Inhibition of CacyBP/SIP phosphorylation can also reduce p38MAPK activity in euESCs. This regulation of the S100A6/CacyBP/SIP/p38MAPK axis may be a promising therapeutic target [147]. In euESCs, S100A6 and β-catenin are expressed in a synergistic manner [148,149]. β-catenin has the potential to become a target for treatment. The expression of miR-202-3p is downregulated in endometriotic tissue. MiR-202-3p targets YAP1 (Yes-associated protein 1) to reduce the expression of S100A6 mediated by STAT3 (signal transducer and activator of transfer 3), thus preventing endometriosis progression [150]. S100A6-related molecular mechanisms play an important role in the occurrence and development of endometriosis and are hence promising therapeutic targets (Figure 5).

S100A6 and its receptor RAGE may be related to smoking-induced inflammation and oxidative damage in bronchial epithelial cells and lung tissues [35]. S100A6 also regulates the cell cycle of hepatic stellate cells, promotes proliferation, and aggravates liver fibrosis, which may be related to activation of the MAPK/ERK pathway [36]. The level of S100A6 level in the Wharton’s jersey of preeclampsia patients has been shown to be higher than that of the healthy control group, and the post-translational modification was also different from that of the control group. It can also directly interact with lumican, PRELP, and IGFBP-1 and participate in preeclampsia development [151]. The S100A6/Vimentin/PKCθ/NF-kB signaling pathway plays an important role in TgSAG1 (*Toxoplasma gondii* surface antigen 1)-mediated parasite invasion and host immune regulation [37]. S100A6 is a key molecule in the life activities of organisms and is involved in the pathophysiological processes underlying many diseases.

## 7. Considerations and Future Directions

S100A6 is a calcium-binding protein that was first purified from the Ehrlich ascites tumor cells. S100A6 is expressed by a variety of mammalian cells and tissues, such as the skeletal muscle, heart muscle, lung, kidney, spleen, neurons, platelets, and lymphocytes. It is also expressed in lung, colorectal, pancreatic, and liver cancers as well as in other cancers such as melanoma. In addition to being involved in tumor invasion, proliferation, and migration, it is also related to the pathogenesis of other non-neoplastic diseases. S100A6 is closely associated with nervous system diseases, leukemia, cardiovascular diseases, osteoarthritis, and endometriosis.

In summary, S100A6 is a key molecule in the body and is involved in many pathophysiological processes. Its complex molecular mechanisms in many diseases make it a potential therapeutic target. Particularly in tumor diseases, leukemia (MLL-AF4-positive ALL), cardiovascular system diseases (myocardial infarction), and endometriosis, it has ample prospects for becoming a target for clinical treatment. Investigation of the molecular function of S100A6 and its involvement in related diseases, and revealing the molecular mechanisms of S100A6’s function in human diseases, will help increase the understanding of these diseases and improve the diagnosis and treatment methods.

## Data Availability

Not applicable.
